# Insights into momentous aroma dominating the characteristic flavor of jasmine tea

**DOI:** 10.1002/fsn3.3701

**Published:** 2023-09-25

**Authors:** Yueling Zhao, Shunyu Li, Xiao Du, Wei Xu, Jinlin Bian, Shengxiang Chen, Chunlei He, Jingyi Xu, Shanrong Ye, Dejian Feng, Pinwu Li

**Affiliations:** ^1^ Department of Tea Science, College of Horticulture Sichuan Agricultural University Chengdu China; ^2^ Tea Refining and Innovation Key Laboratory of Sichuan Province Sichuan Agricultural University Chengdu China; ^3^ National Institute of Measurement and Testing Technology Chengdu China

**Keywords:** aroma profile, correlation analysis, fresh and lovely, green aroma, jasmine tea, quantitative evaluation

## Abstract

Jasmine tea is loved by most people who drink flower tea owing to its unique aroma, and it is known as the top of flower teas. In our study, the quantitative evaluation of the quality of jasmine tea and detection of aroma components were carried out. First, the flavor quality of 92 kinds of jasmine tea was evaluated using multiple sub‐factor quality evaluation methods. According to the evaluation results, jasmine tea was divided into three types: “fresh and lovely” (FL), “heavy and thick” (HT), and “fresh and heavy” (FH). Gas chromatography–mass spectrometry (GC–MS) was used to detect the aroma components of the three types of jasmine tea samples. α‐Farnesene, cis‐3‐hexenyl benzoate, acid phenylmethyl ester, linalool, methyl anthranilate, and indole were the main substances that constituted the basic aroma quality characteristics of jasmine tea. Compared to the FL type, the HT and FH types were weaker in the diversification of the characteristic aroma and accumulation of green, herb, sweet, and roast aroma substances. Green and herb aromas play crucial roles in the fresh and persistent qualities of the three types of jasmine tea, which are the key quality factors research focus of jasmine tea.

## Graphical abstract

Six components, a‐farnesene, cis‐3‐hexenyl benzoate, acetic acid phenylmethyl ester, linalool, methyl anthranilate, and indole, were considered to constitute important components of jasmine tea aroma quality.“Fresh and lovely” type of jasmine tea was richer in the diversity of characteristic aroma than the “heavy and thick” and “fresh and heavy” types of jasmine tea. Green and herb aroma played crucial roles in “fresh” and “persistence” quality of jasmine tea.

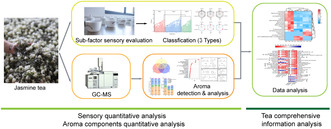

## 1 INTRODUCTION

Jasmine tea is the most popular type of reprocessed tea among consumers. The main production areas are Sichuan, Yunnan, Guangxi, and Fujian. Jasmine tea has attracted people's attention owing to its pleasant aroma. Its aromatic substances can be used in aromatherapy to cure anxiety and tension, where it has a sedative effect (Zhang, Huang, et al., 2021). Currently, the researches on jasmine tea mainly focus on the optimization of the scenting process, influence of the main factors of scenting (the flower amount and scenting time) on quality and their interaction (An et al., 2022; Zhang et al., 2022), optimization of extraction methods for the aroma components of jasmine tea (Ye et al., 2015), and determination of component content (Wang, Zhao, et al., 2020), and there is a lack of research on more effective and accurate evaluations of the sensory and chemical qualities of jasmine tea in the market.

At present, sensory evaluation of jasmine tea focuses mainly on two aspects: the external shape and internal quality (internal qualities, including aroma, liquor color, taste, and leaf base). Their sensory qualities are evaluated by both scoring and review, especially the aroma quality (An et al., 2022). The sensory evaluation of jasmine tea is more complex than that of black or green tea. For a more accurate evaluation of the quality of jasmine tea, the “double cup method” and “twice brewing method” are often used in evaluation (Gong, 2001). Thus, considerable requirements are necessary for the evaluation of tea evaluators. Among them, the aroma and taste quality account for a large proportion (65% of the total score) (China, 2018). However, several problems exist when evaluating, such as absence of detailed evaluation indicators, unclear definitions of some evaluation terms, and confusing meanings of terms (Zhang et al., 2019). Consequently, tea tasters are vulnerable to their own perceptions and external influences, thereby rendering the accuracy of the sensory evaluation results of jasmine tea poor.

The aroma of jasmine tea is caused by a mixture of different volatile compounds with different properties, which are mainly formed by absorbing aroma substances released when jasmine flowers are in bloom (Wang et al., 2008). To date, more than 70 kinds of aroma components have been determined, and their contents account for 0.06%–0.40% of the dry weight of tea, which is the highest among all kinds of tea (Wan, 2003). Research on tea aroma component extraction has been considerably improved (Zhai et al., 2022). Research studies have preliminarily explored the aroma quality of jasmine tea by combining its senses and components and discovered that linalool with floral aroma, methyl anthranilate with grape‐like aroma, 4‐hexanolide with sweet aroma, 4‐nonanolide with sweet aroma, (E)‐2‐hexenyl hexanoate with green aroma, and 4‐hydroxy‐2,5‐dimethyl‐3(2H)‐furanone with sweet aroma were potent odorants in jasmine green tea (Ito et al., 2002). Additionally, the relationship between the aroma components and sensory evaluation requires further analysis. Hence, it is necessary to integrate the results of the instrumental analysis and sensory evaluation data to obtain comprehensive flavor information on jasmine tea.

In this study, the flavor characteristics of jasmine tea were subdivided. By using multi‐level (“factor” → “subfactor”) quality quantitative evaluation methods, the characteristics of the flavor quality of jasmine tea in the market (mainly produced in Sichuan, Yunnan, and other places) were clarified and classified, which has certain reference value for guiding the production direction of jasmine tea. By identifying the key aroma components of the different types of jasmine tea by gas chromatography–mass spectrometry (GC–MS) and studying the correlation between the components and senses, this study provides a theoretical basis for carrying out a systematic evaluation of the quality of jasmine tea and designing its flavor.

## 2 MATERIALS AND METHODS

### 2.1 Instrumentation and equipment

GC–MS: Agilent 7890A/5975C‐GC/MSD, SPME fiber for headspace solid‐phase microextraction (HS‐SPME): 50/30 μm DVB/CAR/PDMS (Sigma‐Aldrich), and gas chromatography column: DB‐5MS (30 m × 250 μm × 0.25 μm) capillary column (Agilent).

### 2.2 Preparation of jasmine tea samples

To evaluate the quality of jasmine tea, 92 samples of green jasmine tea from the main production areas of jasmine tea (Sichuan, Yunnan, etc.) were obtained through market purchases and tea factory sampling in 2020, which were representative. The collected tea samples were coded. Each sample weighed more than 150 g, was packaged in a composite aluminum foil bag, and stored at −5°C. To further obtain the aroma quality data of the different types of jasmine tea, nine representative samples were selected from the three types of jasmine tea: “fresh and lovely” (FL) type (FL1, FL2, FL3), “heavy and thick” (HT) type (HT1, HT2, HT3), and “fresh and heavy” (FH) type (FH1, FH2, FH3).

### 2.3 Sensory quantitative analysis of flavor sub‐factors of jasmine tea

Sensory evaluations of jasmine tea were performed to analyze its sensory quality characteristics. The collected jasmine tea samples were reviewed, and the evaluation group comprised seven senior tea evaluators (three males and four females, average age 32 years) who have approved the tea assessment team report. The sensory room was maintained in a professional tea sensory room under an average daily temperature of 20–25°C and humidity of no more than 70%, with illumination level of about 1000 lx. The quality of the dry tea aroma was assessed as follows: preheat the evaluation cup with boiling water for 5 min and pour it out, and then add 3 g jasmine tea dry tea sample. Next, shake the evaluation cup and sniff the aroma. The quality of the tea taste was assessed according to the Methodology of Sensory Evaluation of Tea (GB/T 23776‐2018) (China, 2018). Simultaneously, to further explore the flavor (aroma and taste) of jasmine tea, the flavor factors were refined (Table S1), and the tea samples were evaluated using the sensory quantitative evaluations of flavor sub‐factors. Each sample was subjected to a password review by evaluators, which was repeated thrice. When calculating the scores of the tea samples, the average score of each item was calculated by removing both the highest and lowest scores of the tea evaluators.

The sensory quantitative scoring standard of the jasmine tea flavor sub‐factor was graded as follows: 0 = odorless, 2 = just perceptible, 4 = weak aroma, 6 = strong aroma, and 8 = extremely strong fragrance.

### 2.4 Construction of jasmine tea aroma contour map

To visually depict the aroma quality characteristics of jasmine tea, radar charts were used to construct the aroma contour maps of jasmine tea based on the clustering analysis results, and the differences in the aroma quality characteristics of the different types of jasmine tea were visually compared. The radar map, also known as the “spider map,” is a two‐dimensional chart that represents multiple variable data on multiple axes, which start at the same point. In this experiment, the aroma sub‐factors were used as the axes, and the distance from the center was used to represent the score of each sub‐factor.

### 2.5 Jasmine tea aroma analysis using GC–MS

For the extraction of aroma components from jasmine tea, aroma components were extracted using HS‐SPME (50/30 μm DVB/CAR/PDMS). 1.0 g tea sample was placed in a 15 mL extraction bottle, and 10 μg ethyl decanoate (10 mg/kg) was used as the internal standard. The extraction bottle was balanced in a constant temperature water bath at 80°C for 30 min and then inserted into SPME fiber (depth: 2 cm) for aroma enrichment for 30 min.

Regarding GC detection conditions, the DB‐5MS (30 m × 250 μm × 0.25 μm) capillary column was applied with pure helium at a flow rate of 1.0 mL/min in the splitless injection mode. The temperature of the GC injector was maintained at 250°C. The column temperature program was as follows: the initial temperature was 60°C (held for 0 min), which further increased to 180°C at 5°C/min (held for 1 min), and to 240°C at 20°C/min (held for 2 min).

Regarding MS detection conditions, the ion source was an electron impact ionization source (EI source), ion source temperature was 230°C, ionization voltage was 70 eV, and mass scanning range was 35–500 u.

The compounds were screened by double comparison of NIST (https://www.nist.gov) standard spectral library and relative retention Index (RI). The RI of each compound was calculated after analyzing C_7_–C_40_ n‐alkane series under the same chromatographic conditions.

As regards the aroma component content, the mass concentration of volatile compounds was calculated with reference to the internal standard method (assuming an absolute correction factor of 1.0 for volatile matter), using Equation (1):

(1) $$C_{i}=\frac{S_{i}}{S_{A}}\times C_{A}$$

where *C*_*i*_ represents the content of aroma substances (mg/kg), *C*_*A*_ is the content of standard samples (mg/kg), *S*_*i*_ is the peak area of aroma substances, and *S*_*A*_ is the peak area of the standard samples (Ayseli et al., 2021).

### 2.6 Aroma contributions of jasmine tea volatile components

The relative odor activity values (ROAVs) are frequently used to evaluate the contribution of aromatic compounds. The greater the ROAV value, the greater the contribution of the volatile components to the aroma of jasmine tea. Compounds with ROAV >1 were considered active aroma compounds, which considerably contributed to the aroma characteristics. The ROAV values were calculated using Equation (2):

(2) $$\text{ROAV}=\frac{C_{i}}{{\mathrm{OT}}_{i}}$$

where *C*_*i*_ is the aroma substance content (mg/kg) and OT_*i*_ is the odor threshold of the corresponding component in water (mg/kg).

### 2.7 Statistical analysis

Excel 2019 was used to collate the experimental data. The tests were repeated thrice, and the results of each test were expressed as the average of the three replicates. SIMCA14.1 software (Umetrics) was used for partial least‐squares discriminant analysis (PLS‐DA), and the partial least‐squares discriminant analysis variables important in the projection (VIP). Origin 8.0 software (Origin Lab) was used for hierarchical clustering analysis, while Spearman correlation analysis and the plotting of Venn diagrams and heat maps were used to visualize the experimental data. The descriptive words for the aroma components were obtained from the flavor information database (https://www.femafLavor.org/) published by the American Essence Flavor Manufacturers Association (FEMA).

## 3 RESULTS AND DISCUSSION

### 3.1 Sensory quality evaluation and flavor classification of jasmine tea

**FIGURE 1:**
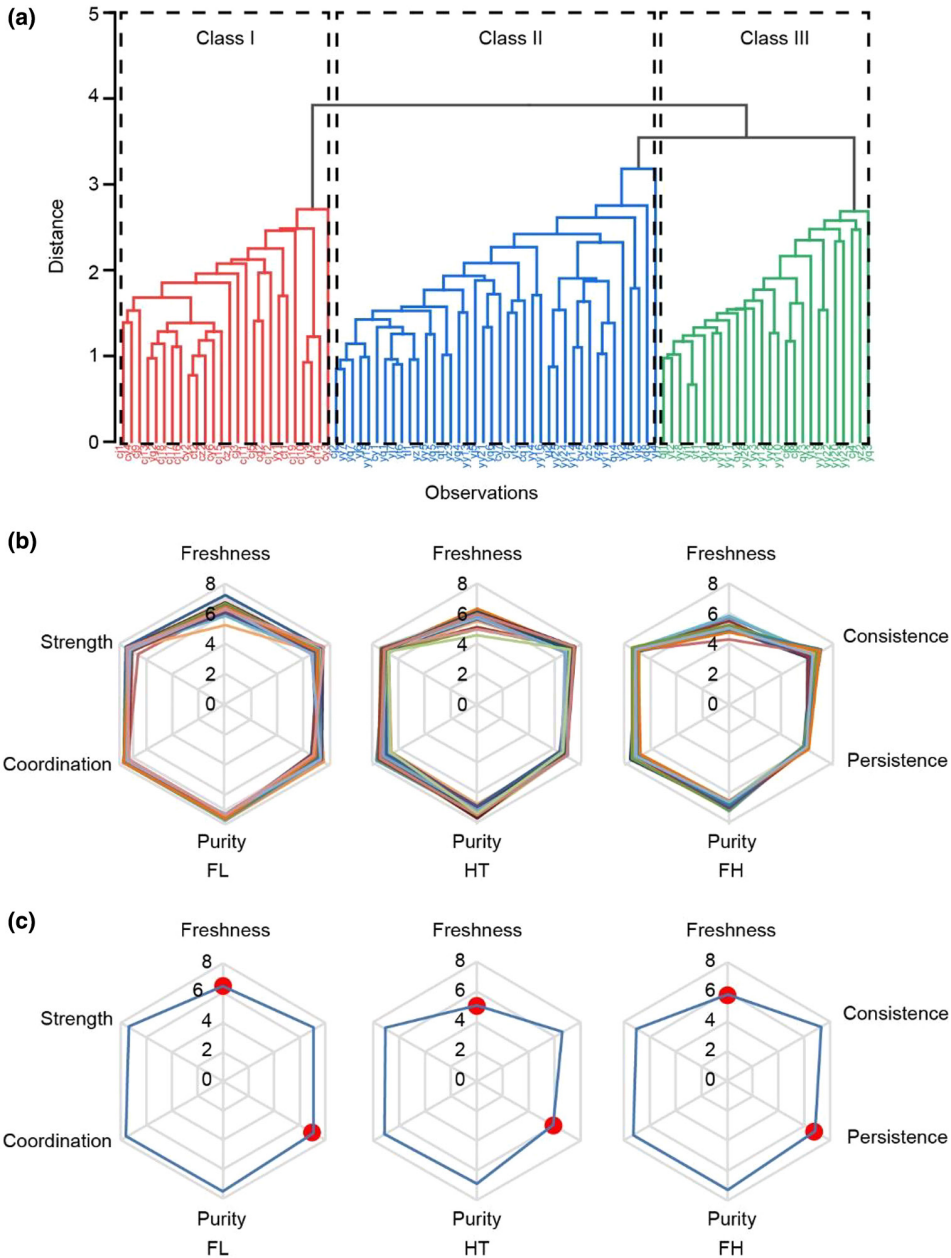
Sensory quality evaluation and flavor classification of jasmine tea. (a) Cluster analysis of the evaluation results of 92 jasmine tea samples. (b) Aroma radar maps of 92 jasmine tea samples. The axis is the score of the indicated aroma sub‐factors. (c) Aroma radar maps of three types of jasmine tea. The axis is the average score of the indicated aroma sub‐factors of the different types of jasmine tea. Red dot, significant differences among the three types of jasmine tea.

Aroma and taste are crucial factors in sensory quality evaluations, which affect the marketable value of jasmine tea (Wang et al., 2022; Zhao et al., 2020). The aroma of dry tea leaves is often the first impression of tea by tea drinkers. To quantitatively evaluate the aroma and taste of jasmine tea, we identified the flavor types of high‐quality jasmine tea. The sensory qualities of 92 commercial kinds of jasmine green teas were evaluated using the sub‐factor sensory quantitative evaluation method (Table S1) from the key aroma and taste items, the results of which are shown in Table S2. The cluster analysis of the evaluation results (Figure 1a and Figure S1) showed that 92 high‐quality jasmine tea samples were grouped into Class I (26, 28.26%), Class II (40, 43.48%), and Class III (26, 28.26%), which shows that high‐quality jasmine tea in the market was mainly divided into three groups.

According to the quality characteristics of the three types of jasmine tea (Table S3), the first type of jasmine tea showed the quality characteristics of fresh fragrance, fresh and pure taste, and obvious flower fragrances. Hence, it was named “fresh and lovely type” (FL). The taste quality of Class II jasmine tea was characterized by an outstanding concentration and thickness in taste quality (Table S4). It was named “heavy and thick type” (HT). The freshness and thickness of the fragrance in Class III jasmine tea were excellent, so it was named “fresh and heavy type” (FH).

To further reflect on the flavor characteristics of the different types of jasmine tea and the differences between them, we constructed aroma profile maps for the different types of jasmine tea to visually describe the fragrance quality of the three types of jasmine tea (Figure 1b). The scores of the “freshness,” “concentration,” and “purity” of the FL jasmine tea in terms of the aroma were high and similar (6.35–7.5), which showed that the aroma of the FL jasmine tea was harmonious. Through the analysis of sample specifications, it was found that this may be because the raw materials of this type of jasmine tea are tender and the number of scenting processes is more. The aroma of the FH jasmine tea was slightly insufficient in terms of the “freshness” which may be because although this kind of jasmine tea mostly goes through the process of separation of flower from tea, the tenderness of the raw material tea is lower than that of FL jasmine tea.

As shown in Figure 1c, there was a slight difference in the quality of the three types of jasmine tea in terms of the consistence, purity, coordination, and strength. Hence, it is believed that these four factors are essential for the flavor quality of jasmine tea. However, the aroma quality of the three types of jasmine tea differed in terms of freshness and persistence factors (*p* < .05). The aroma of the FL jasmine tea was excellent as regards “freshness,” and its score was considerably higher than that of the other two types of jasmine tea. This may be because the more tender the raw materials of the green tea were, the more they contained terpene compounds. Terpene compounds are easier to curl and form during rolling and other molding processes, thereby producing more pore layers and a stronger adsorption capacity. This is conducive to absorbing more aroma substances when separating the flower from tea (Xu et al., 1998) and making the jasmine tea more “fresh and lovely.” The “freshness” and “persistence” scores of the aroma of the HT jasmine tea were lower than those of the other aroma sub‐factors, which is because this type of jasmine tea used more stir‐frying technology after the scenting process. The tea and jasmine flowers were dried together so that the dried jasmine flowers could impact the aroma of the tea, thereby resulting in a great loss of the aroma's freshness. Compared to the other two types of jasmine tea, the “freshness” and “persistence” of the FH jasmine tea were between that of the FL and HT. This is because the raw material tenderness of the FH jasmine tea was lower than that of the FL jasmine tea.

### 3.2 Detection of volatile compounds in jasmine tea using GC–MS

**FIGURE 2:**
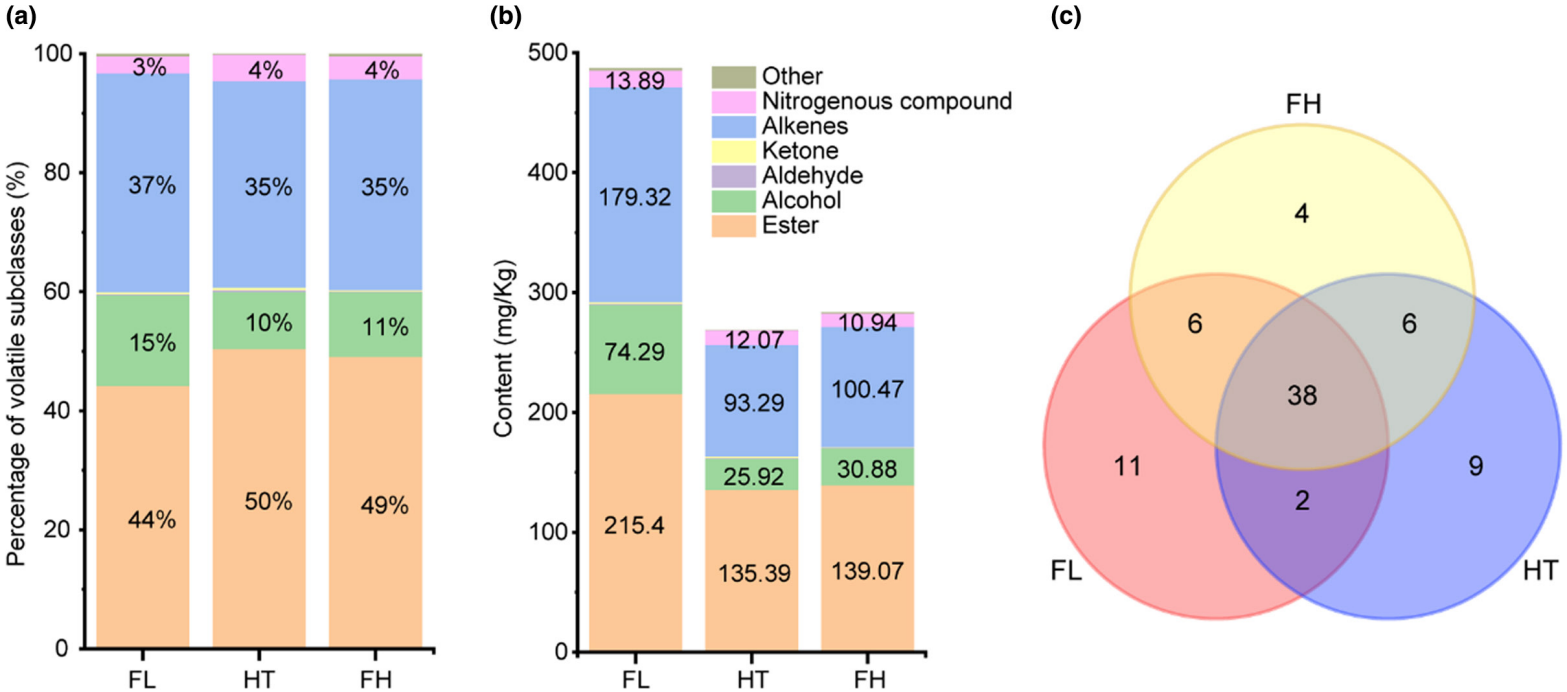
Layout of volatile compounds in three types of jasmine tea. Percentage (a) and content (b) of major classes of volatile substances in the three types of jasmine tea. (c) Venn diagram of aroma compounds in the three types of jasmine tea.

The aroma of tea is a comprehensive reflection of its aroma components by the senses, and the aroma components are the material basis for aroma formation. HS‐SPME/GC–MS was used to detect the aroma components and their contents in the representative samples of the three types of jasmine tea and to explore the aroma material. In the present study, 57, 55, and 54 volatile compounds were identified under the FL, HT, and FH jasmine teas, respectively (Table 1). All the compounds were divided into esters (28), alcohols (13), aldehydes (8), ketones (3), alkenes (20), nitrogenous compounds (2), and others (2), as shown in Figure 2a.

**TABLE 1:** Volatile compounds identified in FL, HT, and FH jasmine tea.

No.	Compound	Description series	RT	CAS	RIcal	RIref	Concentration (mg/kg)		
							FL	HT	FH
Alcohol									
1	(E)‐3‐Hexen‐1‐ol	Green	6.788	928‐97‐2	849.1847892	849	0.93 ± 0.15	0.44 ± 0.11	0.58 ± 0.11
2	Benzyl alcohol	Boiled Cherries, Moss, Roasted Bread, Rose	12.1529	100‐51‐6	1033.929949	1039	8.84 ± 2.55	4.23 ± 0.4	3.42 ± 0.31
3	Trans‐linalool oxide (furanoid)	Floral	13.3054	34995‐77‐2	1073.617324	1086	0.17 ± 0.03	–	–
4	Linalool	Coriander, Floral, Lavender, Lemon, Rose	14.2087	78‐70‐6	1104.723272	1104	53.69 ± 2.73	15.64 ± 0.76	22.35 ± 2.64
5	Phenethyl alcohol	Fruit, Honey, Lilac, Rose, Wine	14.5502	60‐12‐8	1160.757369	1140	0.25 ± 0.01	0.27 ± 0.07	0.21 ± 0.07
6	Linalool oxide III (pyran)	Flower	16.4232	14049‐11‐7	1180.981573	1183	–	0.08 ± 0.03	–
7	Linalool oxide II (pyran)	/	16.4251	39028‐58‐5	1181.047001	1183	0.13 ± 0.04	–	0.07 ± 0.02
8	Nerol	Floral, Fruit	17.8747	106‐25‐2	1230.965282	1225	0.06 ± 0.04	–	–
9	Geraniol	Geranium, Lemon Peel, Passion Fruit, Peach, Rose	18.5757	106‐24‐1	1255.104848	1251	0.59 ± 0.03	0.37 ± 0.04	0.37 ± 0.07
10	Nerolidol	Fir, Linoleum, Pine	26.6489	7212‐44‐4	1551.289025	1567	4.01 ± 0.18	2.36 ± 0.85	1.87 ± 0.02
11	τ‐Cadinol	/	28.5673	5937‐11‐1	1621.964799	1625	2.96 ± 0.87	1.32 ± 0.49	1.21 ± 0.06
12	(E)‐α‐Cadinol	/	28.8554	481‐34‐5	1632.578691	1650	2 ± 0.34	1.21 ± 0.27	0.8 ± 0.06
13	Farnesol	Oil	29.8379	4602‐84‐0	1668.774974	1695	0.66 ± 0.09	–	–
Ketone									
1	6‐Methyl‐5‐hepten‐2‐one	Citrus, Mushroom, Pepper, Rubber, Strawberry	10.6161	110‐93‐0	981.008858	982	1.48 ± 0.1	–	–
2	trans‐β‐Ionone	Floral, Violet	24.6199	79‐77‐6	1476.538636	1478	–	–	0.55 ± 0.09
3	beta‐Ionone	Floral, Violet	24.6229	14901‐07‐6	1476.649159	1975	–	1.18 ± 0.24	–
Aldehyde									
1	Hexanal	Apple, Fat, Fresh, Green, Oil	5.4222	66‐25‐1	802.152237	800	0.08 ± 0.03	–	–
2	Benzaldehyde	Bitter Almond, Burnt Sugar, Cherry, Malt, Roasted Pepper	9.9881	100‐52‐7	959.3831128	959	0.32 ± 0.04	0.18 ± 0.03	0.16 ± 0.05
3	Isovaleraldehyde	Fruit	10.6984	590‐86‐3	983.8429326	949	–	0.02 ± 0.01	–
4	2,6‐Dimethyl‐5‐heptenal	Fruit, Green, Melon	12.7594	106‐72‐9	1054.815322	1058	0.12 ± 0.04	0.09 ± 0.02	–
5	Dodecanal	Citrus, Fat, Lily	12.9119	112‐54‐9	1060.066797	1402	–	0.04 ± 0.02	–
6	Nonanal	Fat, Floral, Green, Lemon	14.3332	124‐19‐6	1109.010542	1108	0.19 ± 0.03	0.17 ± 0.06	0.14 ± 0.04
7	Decanal	Floral, Fried, Orange Peel, Penetrating, Tallow	17.3358	112‐31‐2	1212.407775	1205	0.08 ± 0.01	0.07 ± 0.01	0.06 ± 0.03
8	β‐cyclocitral	/	17.7049	432‐25‐7	1225.118066	1218	0.06 ± 0	0.07 ± 0.02	0.07 ± 0.02
Ester									
1	(Z)‐3‐Hexen‐1‐ol acetate	Green	11.2537	3681‐71‐8	1002.965188	1016	–	–	0.86 ± 0.18
2	4‐Hexen‐1‐ol acetate	/	11.2548	72237‐36‐6	1003.003067	‐	1.65 ± 0.7	–	–
3	(E)‐3‐Hexen‐1‐ol acetate	Fruit	11.2553	3681‐82‐1	1003.020285	1014	–	0.63 ± 0.1	–
4	Formic acid phenylmethyl ester	Fruit	13.4527	104‐57‐4	1078.689732	1079	0.08 ± 0.02	–	–
5	Benzoic acid methyl ester	Herb, Lettuce, Prune, Violet	14.0009	93‐58‐3	1097.567492	1098	6.91 ± 1.24	1.72 ± 0.2	2.61 ± 0.2
6	Acetic acid phenylmethyl ester	Fruit	16.0779	140‐11‐4	1169.090857	1162	76.07 ± 2.28	45.18 ± 1.84	53.41 ± 4.19
7	Methyl salicylate	Almond, Caramel, Peppermint, Sharp	16.9261	119‐36‐8	1198.299387	1197	7.14 ± 2.11	2.63 ± 0.25	3.2 ± 0.32
8	Benzoic acid ethyl ester	Camomile, Celery, Fat, Flower, Fruit	16.289	93‐89‐0	1176.360275	1170	0.44 ± 0.13	0.13 ± 0.03	0.17 ± 0.04
9	cis‐3‐Hexenyl‐α‐methylbutyrate	Fruit	18.021	53398‐85‐9	1236.003254	1232	0.17 ± 0.02	0.1 ± 0.03	0.11 ± 0.02
10	cis‐3‐Hexenyl isovalerate	/	18.1509	35154‐45‐1	1240.476477	1238	0.22 ± 0.04	0.08 ± 0.03	0.11 ± 0.06
11	Hexanedioic acid dimethyl ester	Nuts	18.3016	627‐93‐0	1245.665967	1246	–	0.05 ± 0.03	–
12	2,6‐Dimethyl‐4‐phenyl‐3,5‐pyridinedicarboxylic acid diethyl ester	/	18.4306	1539‐44‐2	1250.108199	1250	–	0.02 ± 0.01	0.02 ± 0
13	Acetic acid‐2‐phenylethyl ester	Flower, Honey, Rose	18.677	103‐45‐7	1258.593204	1258	0.24 ± 0.03	0.24 ± 0.05	0.23 ± 0.07
14	Ethyl salicylate	/	19.0903	118‐61‐6	1272.825562	1272	0.16 ± 0.02	0.06 ± 0.02	0.06 ± 0.01
15	Methyl anthranilate	Flower, Honey, Peach	21.0881	134‐20‐3	1346.423803	1347	27.27 ± 1.6	22.63 ± 1.79	19.97 ± 0.53
16	(E)‐3,7‐Dimethylocta‐2,6‐dien‐1‐yl 3‐methylbutanoate	Apple, Fruit, Rose	22.0338	109‐20‐6	1381.264126	1379	–	1.32 ± 0.26	–
17	Geranyl acetate	Lavender, Rose	22.0342	105‐87‐3	1381.278862	1386	–	–	1.56 ± 0.1
18	cis‐3‐Hexenyl cis‐3‐hexenoate	Green	22.1962	61444‐38‐0	1387.247104	1391	2.6 ± 0.41	1.42 ± 0.36	2.28 ± 0.3
19	cis‐3‐Hexenyl benzoate	Floral	26.924	25152‐85‐6	1561.423984	1574	84.55 ± 4.93	55.32 ± 3.67	51.1 ± 2.93
20	E‐2‐Hexenyl benzoate	/	27.2304	76841‐70‐8	1572.712066	1588	1.65 ± 0.28	–	1.02 ± 0.22
21	cis‐3‐Hexenyl salicylate	/	29.1131	65405‐77‐8	1642.072617	1698	0.52 ± 0.07	0.23 ± 0.18	0.18 ± 0.04
22	Benzyl Benzoate	Balsamic, Herb, Oil	31.3976	120‐51‐4	1785.03135	1785	1.03 ± 0.31	0.73 ± 0.04	0.39 ± 0.06
23	(E)‐2‐Methyl‐propanoic acid‐3,7‐dimethyl‐2,6‐octadienyl ester	Fruit	22.0384	2345‐26‐8	1381.433594	1380	2.84 ± 0.55	–	–
24	2‐(Methylamino)‐benzoic acid methyl ester	Floral, Must	22.8087	85‐91‐6	1409.812217	1412	1.09 ± 0.06	0.59 ± 0.03	0.59 ± 0.04
25	Dihydroactindiolide	Fruit	25.8518	15356‐74‐8	1521.923064	1535	–	0.52 ± 0.07	0.41 ± 0.06
26	Benzoic acid, hexyl ester	Savory	27.063	6789‐88‐4	1566.544883	1576	2.22 ± 1.08	1.19 ± 0.48	1.14 ± 0.47
27	Geranyl valerate	Fruit, Rose	29.5045	10402‐47‐8	1656.492185	1658	–	0.06 ± 0	–
28	N‐Benzoylglycine ethyl ester	/	30.1795	1499‐53‐2	1746.828933	–	0.2 ± 0.11	0.12 ± 0.04	–
Alkenes									
1	β‐Myrcene	Balsamic, Fruit, Geranium, Herb, Must	10.8064	123‐35‐3	987.5620098	994	0.27 ± 0.07	0.11 ± 0.04	0.14 ± 0.06
2	β‐Ocimene	Floral	12.553	13877‐91‐3	1047.707753	1048	0.21 ± 0.05	0.09 ± 0.03	0.12 ± 0.05
3	Alloocimene	/	15.0453	7216‐56‐0	1133.532346	1132	0.09 ± 0.02	–	–
4	α‐Cubebene	/	21.2711	17699‐14‐8	1353.165495	1353	1.07 ± 0.02	0.41 ± 0.12	0.48 ± 0.21
5	Calarene	/	22.3758	17334‐55‐3	1393.863748	1421	–	0.9 ± 0.92	0.99 ± 0.16
6	Eremophilene	/	22.3816	10219‐75‐7	1394.077426	1406	2.56 ± 0.4	–	–
7	(−)‐Isoledene	/	22.9538	95910‐36‐4	1415.157846	1414	–	0.14 ± 0.03	0.14 ± 0.02
8	l‐Caryophyllene	Fried, Spice, Wood	23.1907	87‐44‐5	1423.885479	1424	1.23 ± 0.2	0.33 ± 0.12	0.39 ± 0.09
9	(+)‐Aromadendrene	/	23.6672	489‐39‐4	1441.440215	1444	0.15 ± 0.04	–	0.36 ± 0.1
10	(E)‐β‐Famesene	Spice, herb, fresh green, sweet	23.9625	18794‐84‐8	1452.319363	1455	1.29 ± 0.44	0.42 ± 0.18	0.44 ± 0.03
11	γ‐Muurolene	/	24.5767	30021‐74‐0	1474.947105	1473	2.44 ± 0.95	–	0.66 ± 0.04
12	α‐Copaene	/	25.0187	3856‐25‐5	1491.230827	1492	–	0.69 ± 0.08	–
13	α‐Muurolene	/	25.1278	31983‐22‐9	1495.25018	1497	3.05 ± 0.46	–	–
14	α‐Farnesene	Boiled Vegetable, Floral, Wood	25.3175	502‐61‐4	1502.238918	1511	152.42 ± 24.96	86.21 ± 4.42	89.47 ± 2.93
15	γ‐Cadinene	/	25.5351	39029‐41‐9	1510.255519	1515	4.47 ± 0.46	2.27 ± 0.37	1.77 ± 0.41
16	δ‐Cadinene	Fruit	25.6539	483‐76‐1	1514.63223	1518	8.9 ± 0.74	–	2.46 ± 0.31
17	Thujopsene	/	25.7274	470‐40‐6	1517.340043	1529	–	1.39 ± 0.34	1.44 ± 0.14
18	α‐Cadinene	/	26.0924	24406‐05‐1	1530.787008	1539	0.89 ± 0.1	0.33 ± 0.04	0.33 ± 0.04
19	α‐Patchoulene	/	26.7599	560‐32‐7	1555.378376	1571	0.28 ± 0.08	–	0.2 ± 0.01
20	β‐Bisabolene	Floral	27.857	495‐61‐4	1595.796637	1547	–	–	1.08 ± 0.07
Nitrogenous compound									
1	Indole	Burnt, Mothball	19.7594	120‐72‐9	1295.866622	1296	13.2 ± 1.37	11.15 ± 0.42	10.54 ± 0.05
2	Caffeine	/	33.1555	58‐08‐2	1840.163138	1840	0.69 ± 0.09	0.92 ± 0.04	0.4 ± 0.02
Other									
1	(Z)‐3‐Hexenyl ester‐butanoic acid	/	16.7117	16491‐36‐4	1190.91633	1188	–	0.42 ± 0.15	0.67 ± 0.18
2	3‐Allyl‐6‐methoxyphenol	/	21.3566	501‐19‐9	1356.315401	1362	0.58 ± 0.03	0.4 ± 0.07	0.29 ± 0.04

*Note*: “–” indicates no data.

Esters, alkenes, and alcohols are the major volatile subclasses of jasmine tea (Figure 2a). Esters are the most abundant classes of volatile compounds in the seven classes of volatiles, accounting for 44%–50% of the identified volatiles. The alkenes (35%–37%) are the second most abundant class of volatile compounds, followed by alcohols (10%–15%), nitrogenous compounds (3%–4%), ketones (0.19%–0.44%), and aldehydes (0.17%–0.24%). Other studies also found similar results in the volatile subclass content of jasmine teas (An et al., 2022). During jasmine‐scented black tea processing, the amount of esters, terpenes, and terpenoids increased greatly, while the amount of aldehydes decreased. Finally, the ester content was the most abundant, which was formed during the scenting process (Li et al., 2018), while aldehydes were the least abundant.

According to the total content of the aroma of the three types of jasmine tea (Figure 2b), the FL jasmine tea (487.38 mg/kg) is greater than the FH jasmine tea (283.68 mg/kg) and the HT jasmine tea (268.92 mg/kg), which can be seen from the test materials that this is directly related to the amount of flowers used in the scenting process of the three types of jasmine tea, because the amount of flowers directly affected the total amount of aroma substances released by jasmine flowers (Fang et al., 2004). Simultaneously, it was also related to the tenderness and ability to absorb the fragrance of the base tea (Xu et al., 1998).

As shown in the Venn map (Figure 2c), the distribution of the volatile profiles of each sample was well clarified. Among them, 38 components were shared by three types of the jasmine tea, which accounts for the majority of the total aroma. This shows that these 38 common components are an integral part of the aroma quality of jasmine tea. Six components, α‐farnesene, cis‐3‐hexenyl benzoate, acetic acid phenylmethyl ester, linalool, methyl anthranilate, and indole, were highly abundant (>10 mg/kg) in the aromatic fractions of the three classes of the jasmine tea. Meanwhile, these six components were mostly considered to constitute the important components of the jasmine tea aroma quality in previous studies (An et al., 2022; Lu et al., 2004; Wang, Zhao, et al., 2020), especially the five components except for acetic acid phenylmethyl ester, which was also defined as the jasmine tea aroma quality evaluation index (JTF index) (Lin et al., 2013).

It can be seen from Figure S2A that there are obvious differences in the content of the six components in the three types of jasmine tea. Regarding the total amount of six components, FL (407.20 mg/kg) > FH (246.84 mg/kg) > HT (236.13 mg/kg), accounting for 87.12%–94.55% of the total amount of 38 common aroma components. It can be perceived that α‐farnesene is a crucial reason for the difference in the common aroma components of the three types of jasmine tea, the average contents of which were 152.42 mg/kg (FL) > 89.47 mg/kg (FH) > 86.21 mg/kg (HT), which were found increased 6.67 times in jasmine‐scented tea compared to the base tea (Zhang et al., 2022) and is also one of the major volatile constituents of several flowers (Edris et al., 2008; Lee et al., 2019). The proportions of the related substances in the three types of jasmine tea were similar (Figure S2B), which suggests that during the FL jasmine tea processing, the absorption of the jasmine fragrance by green tea was several times greater than that of the two other types.

By analyzing the specific aroma components of the three types of jasmine tea (Figure 2c), 11 unique aroma components in the FL jasmine tea, including α‐muurolene (3.05 mg/kg), (E)‐2‐methyl‐propanoic acid‐3,7‐dimethyl‐2,6‐octadienyl ester (2.84 mg/kg), eremophilene (2.56 mg/kg), 4‐hexen‐1‐ol acetate (1.65 mg/kg), 6‐methyl‐5‐hepten‐2‐one (1.48 mg/kg), farnesol (0.66 mg/kg), trans‐linalool oxide (furanoid) (0.17 mg/kg), alloocimene (0.09 mg/kg), hexanal (0.08 mg/kg), formic acid phenylmethyl ester (0.08 mg/kg), and nerol (0.06 mg/kg) were identified. Meanwhile, there are nine specific aroma components in the FH jasmine tea, including (E)‐3,7‐dimethylocta‐2,6‐dien‐1‐yl 3‐methylbutanoate (1.32 mg/kg), α‐copaene (0.69 mg/kg), and (E)‐3‐hexen‐1‐ol acetate (0.63 mg/kg). Only four distinctive aroma components in the HT jasmine tea, including geranyl acetate (1.65 mg/kg), β‐bisabolene (1.08 mg/kg), (Z)‐3‐hexen‐1‐ol acetate (0.86 mg/kg), and trans‐β‐Ionone (0.55 mg/kg). However, the content of the special components of the three kinds of jasmine tea was low, accounting for 1.43%–2.60% of the total aroma of all kinds of jasmine tea.

### 3.3 Screening aroma‐active compounds dominating the characteristic flavor in jasmine tea

**FIGURE 3:**
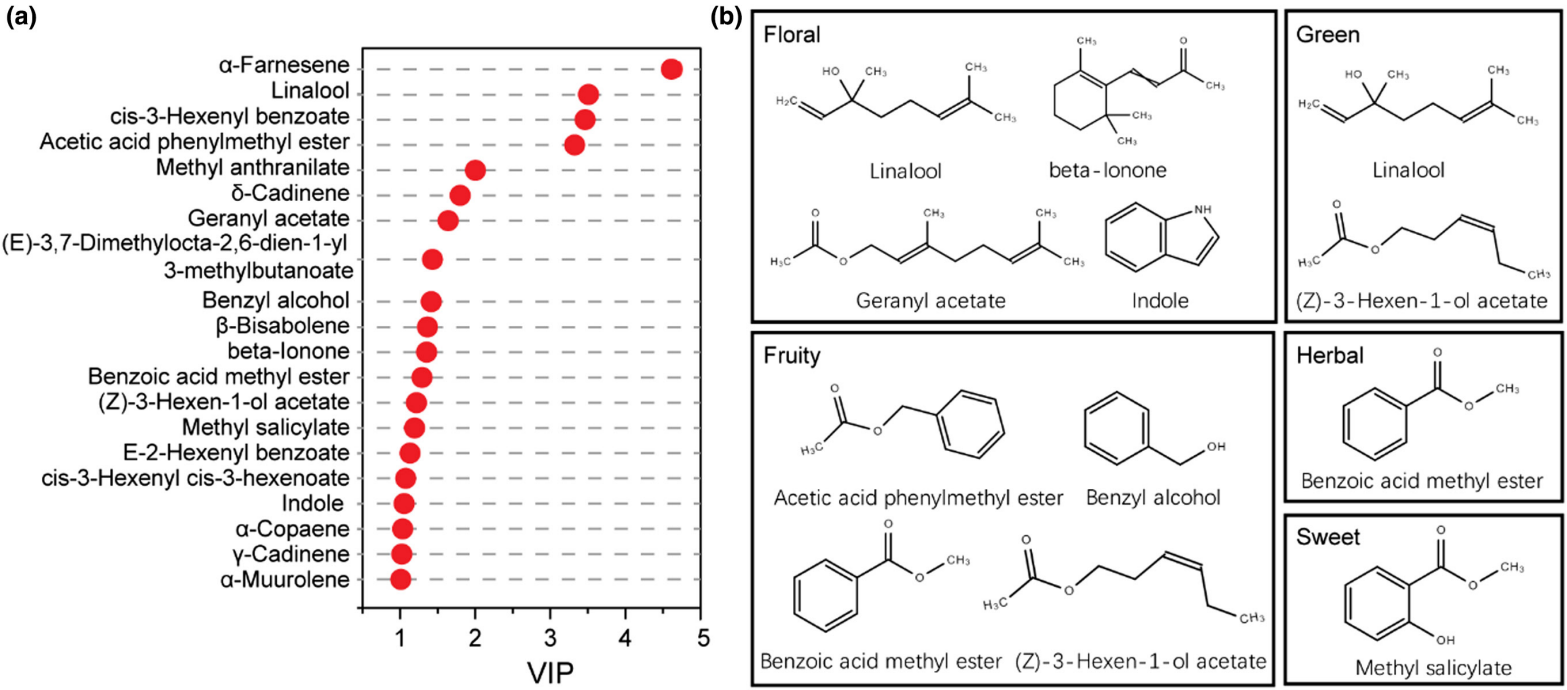
Screening aroma‐active compounds dominating the characteristic fragrances in jasmine tea. (a) The volatile compounds with VIP score >1 in jasmine tea. (b) The aroma‐active compounds with VIP score >1 and ROAV >1.

Moreover, those odor‐contributing volatiles with variable important for the project (VIP) > 1 were sorted out and displayed in Figure 3a. Twenty‐one differential volatile compounds were obtained with VIP >1, including α‐farnesene, linalool, cis‐3‐hexenyl benzoate, acetic acid phenylmethyl ester, methyl anthranilate, δ‐cadinene, geranyl acetate, (E)‐3,7‐dimethylocta‐2,6‐dien‐1‐yl 3‐methylbutanoate, benzyl alcohol, β‐bisabolene, beta‐ionone, benzoic acid methyl ester, (Z)‐3‐hexen‐1‐ol acetate, methyl salicylate, E‐2‐hexenyl benzoate, cis‐3‐hexenyl cis‐3‐hexenoate, and indole. In particular, α‐farnesene (having a boiled vegetable, floral, and wood flavor) had the highest VIP score, followed by linalool contributing a coriander, floral, lavender, lemon, and rose flavor, cis‐3‐hexenyl benzoate contributing a floral aroma, acetic acid phenylmethyl ester contributing a fruity aroma, methyl anthranilate contributing a flower, honey, and peach aroma. However, judging the importance only from the aroma content is inadequate to determine whether the substances contribute to the aroma quality of jasmine tea. Therefore, it is necessary to introduce the ROAV to evaluate the contribution of these aroma components and finally determine the aroma components that cause the special aroma quality of jasmine tea. Consequently, major aroma contributors were further screened based on ROAVs >1, which were calculated according to their aroma thresholds and concentrations (Wang, Hua, et al., 2020; Wang, Ma, et al., 2020; Wang, Zhao, et al., 2020) (Table S5). The ROAV values of linalool, acetic acid phenylmethyl ester, nerolidol, and indole were higher (>100), especially for linalool and acetic acid phenylmethyl ester. Both components are believed to be important substances that constitute the “basic aroma” of the jasmine tea. By comparing the six components with high contents in the three types of jasmine tea, the ROAV values of linalool, acetic acid phenylmethyl ester, and indole were discovered to be all high, showing that these three components were the primary aroma substances of jasmine tea. Whereas, also highly abundant α‐farnesene, cis‐3‐hexenyl benzoate, could not be quantitatively evaluated for their contribution to jasmine tea aroma because the corresponding component thresholds could not be queried. In addition, seven compounds, including benzyl alcohol, linalool, (Z)‐3‐hexenyl acetate ((Z)‐3‐hexen‐1‐ol acetate), methyl benzoate (benzoic acid methyl ester), benzyl acetate (acetic acid phenylmethyl ester), indole, and α‐farnesene, were identified as the primary odor substances in jasmine‐scented tea using weighted gene co‐expression network analysis (WGCNA) (An et al., 2022) and also in jasmine black tea (Li et al., 2018), which were similar to the results of our study.

Consequently, nine were found had the ROAV and VIP over 1.0 and were considered as the characteristic aroma components (Figure 3b). Four floral, four fruity, two green, one herbal, and one sweet odorants were the main characteristic aroma components of jasmine tea (Figure 3b). Combined with the VIP >1 components, these results suggested that those volatiles with high ROAV and VIP values could play an important role in the floral and fruity aroma of jasmine tea. Linalool is the main component of some essential oils from aromatic flora, representing approximately 70% of these volatile concentrates (Knudsen et al., 2006), which emerged as a promising bioactive compound in therapeutic and medicinal tools for depression remedy (dos Santos et al., 2022). Acetic acid phenylmethyl ester is a fragrance constituent which is used in decorative cosmetics, fine fragrances, shampoos, toilet soaps, and other toiletries (McGinty et al., 2012), the ROAV of which was greater than 1 in jasmine tea samples, but less than 1 in green tea (An et al., 2022). Geranyl acetate is a bioactive compound in lemongrass essential oil, which have promising anticancer activities in vitro (Zhang et al., 2022). Benzyl alcohol, with “fruity” and “floral” aromas, was abundant in the different types of tea (Jiang et al., 2021; Ni et al., 2020). β‐Ionone is widely distributed in flowers, fruits, and vegetables (Aloum et al., 2020), and its synthesis ultimately regulates the floral scent of sweet flowers (Han et al., 2019). Benzoic acid methyl ester is a constituent of the floral scent profiles of several flowering plants (Knudsen et al., 2006). (Z)‐3‐hexenyl acetate is a green leaf volatile that serves as a signaling molecule that triggers defense responses (Farag et al., 2005). Methyl salicylate is a crucial marker in grapes and wine, which can induce plant defense resistance (Poitou et al., 2021). Indole is known for its floral odor, which is typical of jasmine blossoms. It affects the jasmine quality (Issa et al., 2020; Mindt et al., 2022) and is an important fragrance compound in tea (Yang et al., 2021; Ye et al., 2021). All those above volatile substances were mostly identified as the key volatile compounds of jasmine flowers or tea, which affected the aroma quality of jasmine tea. Compared to green tea, 2,6,10,10‐tetramethyl‐1‐oxaspiro [4.5] dec‐6ene, linalool, cedrol, 3‐methyl‐butanal, trans‐β‐ionone, and τ‐cadinol were the main odor contributors (Wang, Hua, et al., 2020; Wang, Ma, et al., 2020; Wang, Zhao, et al., 2020). Moreover, combined with above study results, it is speculated that the key fragrance of jasmine tea mainly comes from jasmine flowers, which was intaken by the base tea in the scenting procedure.

### 3.4 Analysis of characteristic aroma compounds among different types of jasmine tea

**FIGURE 4:**
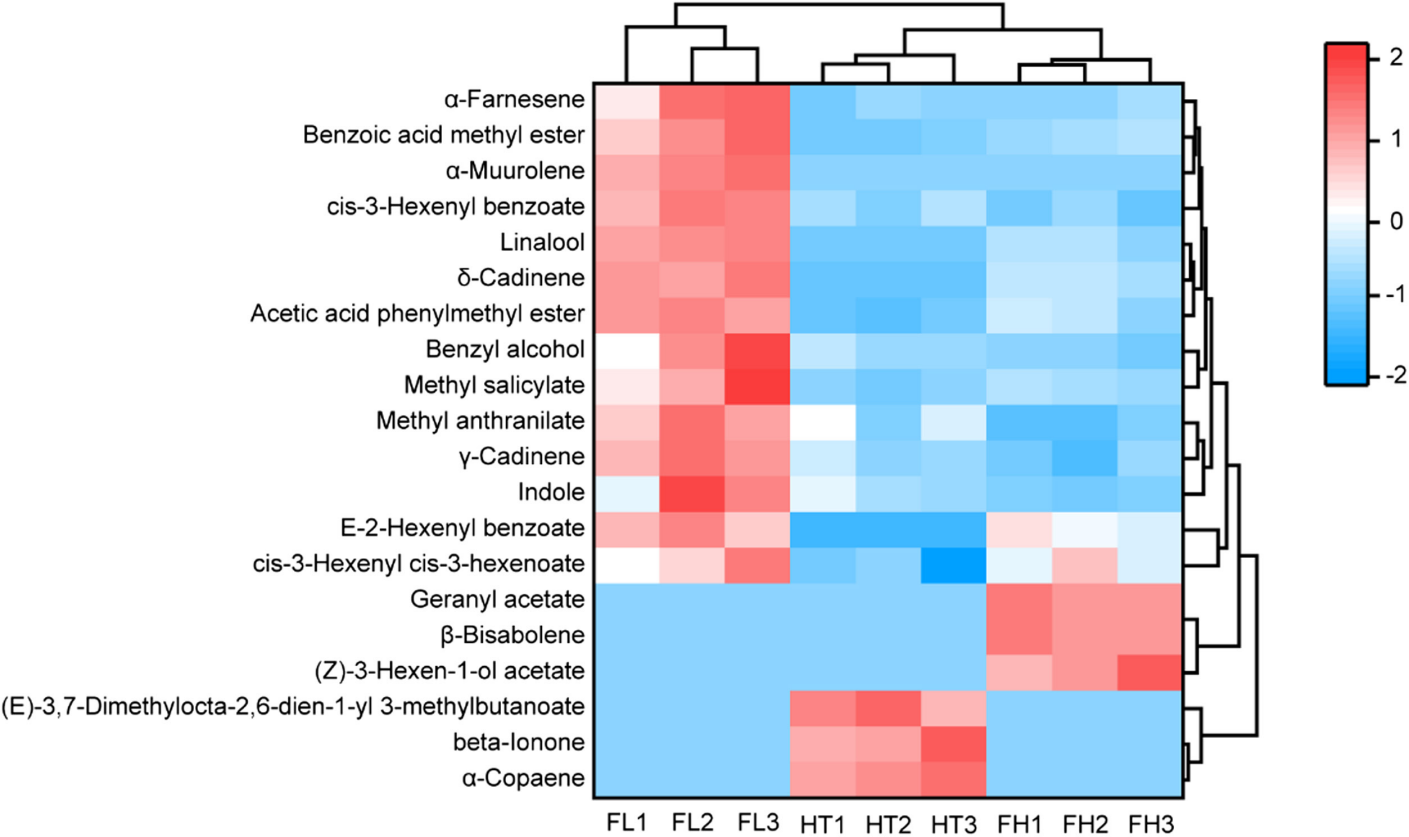
Hierarchical clustering analysis of the key differential aroma‐active compounds among three types of jasmine tea.

Since some aroma substances do not have aroma thresholds (such as cis‐3‐hexenyl benzoate), we conducted a hierarchical cluster analysis on 20 substances with VIP >1 to prevent leakage. As shown in Figure 4, the HT type was closer to the FH type, whereas the FL‐type jasmine tea had unique properties according to the hierarchical cluster analysis. These results were corresponded with the sensory evaluation and aroma component analysis results, which were mutually verified (Figure 1a).

In all the key aromatic active compounds, ester‐derived aromas were the main factors that formed the characteristic flavor of the FL‐type jasmine tea, followed by alkene‐derived aromas. According to the type of aroma, the characteristic aroma components of the FL jasmine tea were mainly floral (α‐farnesene, cis‐3‐hexenyl benzoate, linalool, methyl salicylate, E‐2‐hexenyl benzoate), fruity (benzoic acid methyl ester, δ‐cadinene, acetic acid phenylmethyl ester, methyl salicylate) and sweet (methyl salicylate, methyl anthranilate), coupled with roast (benzyl alcohol, methyl salicylate, indole), herb (benzoic acid methyl ester), and green (cis‐3‐hexenyl cis‐3‐hexenoate) fragrance, leading to the aroma of the FL type more diversified. However, the characterized fragrances of the FH jasmine tea were occupied by ester components and coupled with alcohol, which was identified as floral (E‐2‐hexenyl benzoate, geranyl acetate, β‐bisabolene), fruity ((Z)‐3‐hexen‐1‐ol acetate, (E)‐3,7‐dimethylocta‐2,6‐dien‐1‐yl 3‐methylbutanoate), and green (cis‐3‐hexenyl cis‐3‐hexenoate) fragrances. The HT‐type jasmine tea was distinguished by its fruity ((E)‐3,7‐dimethylocta‐2,6‐dien‐1‐yl 3‐methylbutanoate) and floral (beta‐ionone) fragrances. Compared to the FL type, the HT and FH types of jasmine teas were weaker in the diversity of characteristic aroma and accumulation of green, herb, sweet, and roast aroma substances. The floral and fruity aromatic types were the basal fragrance patterns of jasmine tea. The fragrance diversity of the FL jasmine tea is relatively rich, and the aroma types of FH and HT jasmine teas are relatively single. However, the relationship between the diversity and coordination of the aroma and ratio coefficient of aroma substances required further study. Simultaneously, green and sweet aromatic substances were extracted as potent odorants in Chinese jasmine green tea using the aroma extract dilution analysis (Ito et al., 2002). Additionally, “woody” and “sweet” were applied as the terms for evaluating the odor profile of jasmine tea infusion (Ito & Kubota, 2005).

### 3.5 Correlation analysis between aroma sensory quality and aroma component contribution of jasmine tea

The aroma quality is an extremely important building block of the quality of jasmine tea. Presently, the evaluation of the aroma quality of jasmine tea is mainly conducted using sensory evaluation and chemical component analysis. Further, the relationship between the sensory quality of jasmine tea aroma and its chemical components remains unclear and requires further research. Hence, a correlation analysis between the sensory evaluation score of the proton factor of the jasmine tea aroma and the content (VIP > 1) or ROAV value of the important aroma components in jasmine tea was proposed to explore the relationship between the sensory quality and contribution of aroma components.

**FIGURE 5:**
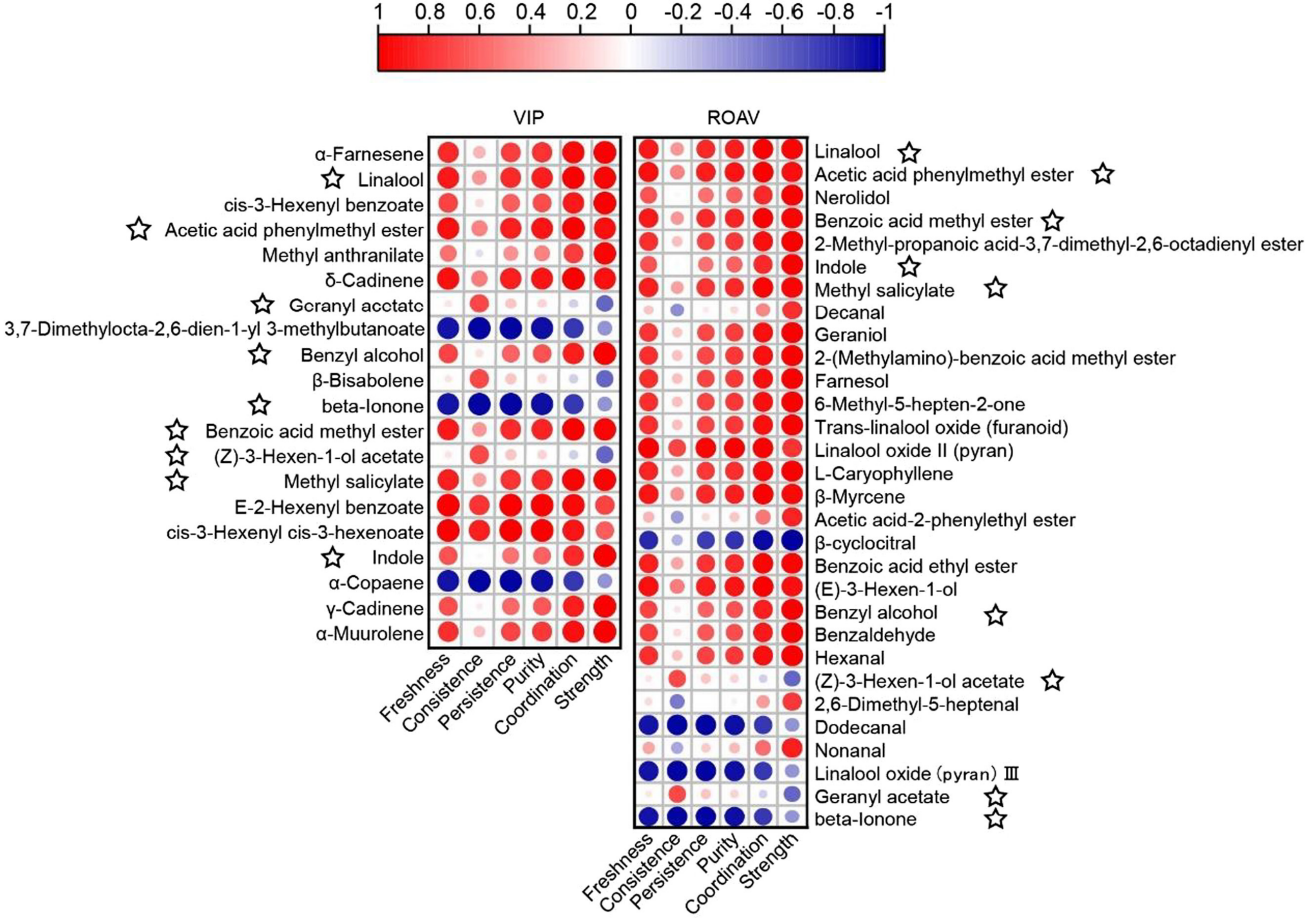
Correlation analysis between aroma sensory quality and component contribution of jasmine tea. Left: compounds with VIP >1; Right: compounds with ROVA >1. Five‐pointed star: the common components on both sides.

As shown in Figure 5, most of the key aroma components contributed considerably, and the correlation coefficients ranged from 0.93 to 0.99. Illustrating that the key aroma components played an important role in the strength of the three types of jasmine teas. Similar to the strength factor, the coordination quality also correlated with several key aroma substances (0.91–0.99). For the freshness quality, the characteristic flavor of the FL jasmine tea, the aroma components acetic acid phenylmethyl ester (fruity), δ‐cadinene, E‐2‐hexenyl benzoate (floral), cis‐3‐hexenyl cis‐3‐hexenoate (green), β‐myrcene (fruity, herb), (E)‐3‐hexen‐1‐ol (green), and linalool oxide II (pyran) highly correlated with the freshness factor, which all positively correlated, while (E)‐3,7‐dimethylocta‐2,6‐dien‐1‐yl 3‐methylbutanoate (floral, fruity), beta‐ionone (floral), α‐copaene, dodecanal (fruity, floral, fat), and linalool oxide (pyran) III (floral) were inversely correlated. This implies that the green and herb aromas played a crucial role in fresh quality. The components of jasmine tea that constituted the “freshness” aroma have always been a hot topic of research. This study showed that the “freshness” of the jasmine tea was a composite aroma comprising different aroma components, while the green and herb aroma in the above results played an important role in the “freshness” of the three types of jasmine tea aroma. Research shows that real and fake jasmine tea can be distinguished according to the disappearance of grass/green smells. Research shows that real jasmine tea (scented with fresh flowers) and fake jasmine tea (scented with aromatic essential oils) can be distinguished according to the disappearance of some grass/green smells (Wang et al., 2008). Further, the herbal scent was found to be related to either the sweet or green scent (Zhang, Huang, et al., 2021; Zhang, Li, et al., 2021). Only a few aromas negatively correlated with consistency factors, such as (E)‐3,7‐dimethylocta‐2,6‐dien‐1‐yl 3‐methylbutanoate (floral, fruity), beta‐ionone (floral), and α‐copaene. Consistency was poorly positively correlated with other contributing components, probably because consistency is a combined reflection of all aroma substances in jasmine tea and is not restricted to a single component. As regards the persistence quality, E‐2‐hexenyl benzoate (floral), cis‐3‐hexenyl cis‐3‐hexenoate (green), and linalool oxide II (pyran) were positively correlated. Finally, positive correlations between acetic acid phenylmethyl ester (fruity), δ‐cadinene, E‐2‐hexenyl benzoate (floral), cis‐3‐hexenyl cis‐3‐hexenoate (green), (E)‐3‐hexen‐1‐ol (green), linalool oxide II (pyran), and sub‐quality purity were detected. Generally, green and herb aromas played a more important role in the differentiation of sub‐factors (fresh and persistent) of the three types of jasmine tea, while the floral and fruity aromas were foundational elements. However, excessive floral and fruity fragrances reduced the holistic effect of the jasmine tea aroma. In the same way, the excessive amount of flowers, scenting time, and scenting temperature were not necessarily conducive to the aroma quality of jasmine tea (An et al., 2022).

Generally, among the three types of jasmine tea, the FL jasmine tea had the highest score in the evaluation and was more popular in the market. We conducted a systematic analysis of the aroma substances of the three types of jasmine tea, showing potential in addressing the issues of the absence of detailed evaluation indicators, unclear definitions of some evaluation terms, and confusing meanings of terms, but more experimental design and in‐depth analysis are necessary to strengthen our findings and make them more scientifically valid. The interaction of volatiles from tea and jasmine flowers significantly modified the properties of their intrinsic olfactory properties. In the process of collision between tea and flowers, how the aroma substances change and interact with each other remains to be further studied.

## 4 CONCLUSION

In the present study, 92 kinds of jasmine tea in the market were first divided into three types (“FL,” “HT,” and “FH” types) using the sensory quantitative evaluation of flavor factors, and the “flavor profile map” of jasmine tea aromas was constructed according to the score of the systematically evaluated sensory quality of the jasmine tea. The content of the aroma components in representative jasmine teas was determined using the HS‐SPME/GC–MS component detection method. The contributions of volatile components to the aroma quality were evaluated by comparing the aroma component content and calculating the ROAV of the typical components. In view of this, the aroma of the jasmine tea was evaluated using chemometrics, and the flavor quality of different types of jasmine tea was explored.

## AUTHOR CONTRIBUTIONS

**Yueling Zhao:** Formal analysis (lead); writing – original draft (lead). **Shunyu Li:** Data curation (equal); investigation (equal). **Xiao Du:** Funding acquisition (equal). **Wei Xu:** Conceptualization (equal); supervision (equal). **Jinlin Bian:** Conceptualization (equal); supervision (equal). **Shengxiang Chen:** Conceptualization (equal); supervision (equal). **Chunlei He:** Conceptualization (equal); supervision (equal). **Jingyi Xu:** Conceptualization (equal); supervision (equal). **Shanrong Ye:** Data curation (equal). **Dejian Feng:** Data curation (equal). **Pinwu Li:** Funding acquisition (equal); writing – review and editing (equal).

## CONFLICT OF INTEREST STATEMENT

The authors declare no competing financial interests.

## ETHICS STATEMENT

In the sensory evaluation part of this study, trained evaluators were used to conduct necessary sensory evaluation on tea samples. All methods were carried out in accordance with relevant guidelines and regulations. Informed consent was obtained from all subjects before their participation in the study.

## Supporting information


Appendix S1

## Data Availability

The data that support the findings of this study are available in the supplementary material of this article.
