# Supplementary material for: Insights into momentous aroma dominating the characteristic flavor of jasmine tea
Source: Food Sci Nutr. 2023 Sep 25;11(12):7841–54. doi: 10.1002/fsn3.3701 (PMC10724623; doi:10.1002/fsn3.3701)
Supplement: Supplementary file 1 — Appendix S1 [file FSN3-11-7841-s001.docx]

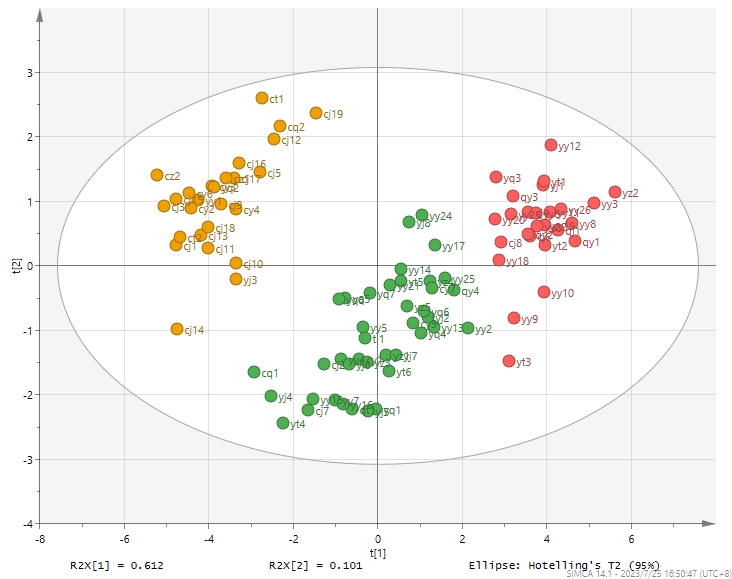


Fig S1. Partial least-squares discriminant analysis score plot of sensory evaluation score in three types of jasmine tea.


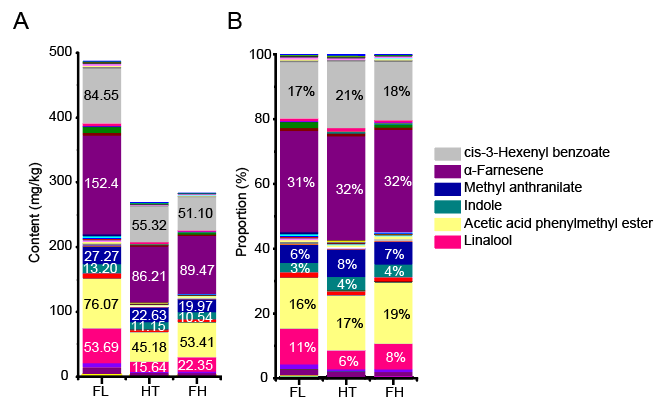


Fig S2. Content (A) and proportion (B) of key volatile substances in three types of jasmine tea.

Table S1 Evaluation index of aroma and taste quality of jasmine tea

| Qaulity | Factor | Subfactor | Paraphrase |
| --- | --- | --- | --- |
| Aroma | Category | Freshness | The freshness of jasmine tea is pleasing to the nose. The freshness of jasmine tea leads to a rich and pleasant floral fragrance, which can be felt at the first smell. |
|  | [Dense](javascript:;)/Light | Consistence | The rich degree of aroma substances: the dense aroma leads to the rich fragrance of flowers. If the fragrance is light, the fragrance of flowers will be weak. |
|  |  | Persistence | Duration of aroma maintenance |
|  | Pure/ Heterologous | Purity | The degree of purity of the aroma: pure means floral and tea aroma was normal, and there was no dissimilarity. |
|  |  | Concentration | Degree of harmony between floral and tea aroma: incongruence leas to tea taste or flower taste is too strong. |
|  | High/Low | Strength | The degree of sensation caused by aroma stimulation. |
|  |  |  |  |
|  | Category | Freshness | Basic taste, fresh and refreshing. |
|  |  | Sweet | Basic taste, sweet taste. |
|  |  | Bitter | Basic taste, with bitter taste in the mouth and bitter aftertaste. |
|  |  | Astringent | After the entrance, there is a feeling of thick tongue blocking and tongue scraping, like eating raw persimmons |
| Taste | [Dense](javascript:;)/Thick | Dense | It contains rich substances and has a strong taste. |
|  |  | Thick | It contains rich substances and has a thick aftertaste. |
|  | Pure/ Heterologous | Purity | The purity of taste. |
|  |  | Coordination | The harmonious degree of the taste of the tea soup after the entrance. |
|  | Floral | Floral | The entrance is fragrant with flowers. |

Note: The "concentration" of aroma in the table refers to the concentration of flower fragrance.

Table S2 Sensory evaluation results of 92 kinds of jasmine tea

| NO. |  |  | Aroma |  |  |  |  |  |  |  |  | Taste |  |  |  |  |
| --- | --- | --- | --- | --- | --- | --- | --- | --- | --- | --- | --- | --- | --- | --- | --- | --- |
|  | Freshness | Consistence | Persistence | Purity | Concentration | Strength |  | Freshness | Sweet | Bitter | Astringent | Dense | Thick | Purity | Coordination | Floral |
| cj1 | 4.88 | 6.41 | 5.78 | 6.74 | 7.03 | 6.93 |  | 3.47 | 3.76 | 4.27 | 3.53 | 6.10 | 5.23 | 6.96 | 6.09 | 2.96 |
| cj2 | 5.66 | 7.22 | 6.62 | 7.03 | 7.00 | 7.10 |  | 5.14 | 3.96 | 3.96 | 3.14 | 5.57 | 5.17 | 7.07 | 6.39 | 4.37 |
| qj1 | 6.53 | 7.13 | 7.06 | 7.60 | 7.37 | 7.33 |  | 5.67 | 4.56 | 2.27 | 1.29 | 5.04 | 4.43 | 7.47 | 7.00 | 6.37 |
| cj3 | 4.21 | 6.53 | 5.90 | 6.82 | 7.00 | 7.00 |  | 3.17 | 4.03 | 4.00 | 3.29 | 6.83 | 5.87 | 7.04 | 6.23 | 2.37 |
| cj4 | 6.28 | 7.53 | 6.90 | 7.32 | 7.53 | 7.38 |  | 5.37 | 4.50 | 3.17 | 1.67 | 5.86 | 3.23 | 7.43 | 6.73 | 6.23 |
| cj5 | 5.19 | 6.63 | 5.82 | 7.10 | 7.41 | 7.10 |  | 3.53 | 4.27 | 4.37 | 3.23 | 6.96 | 5.50 | 7.07 | 7.00 | 3.33 |
| cj6 | 6.28 | 6.81 | 7.19 | 7.47 | 7.37 | 7.13 |  | 5.43 | 4.57 | 2.71 | 1.43 | 4.71 | 4.00 | 7.33 | 7.14 | 5.76 |
| yy1 | 4.88 | 6.38 | 5.74 | 6.94 | 7.01 | 6.90 |  | 3.19 | 3.86 | 3.27 | 3.43 | 6.76 | 5.37 | 7.27 | 5.93 | 3.17 |
| cj7 | 5.82 | 7.06 | 6.66 | 6.94 | 6.84 | 6.74 |  | 4.86 | 3.76 | 4.33 | 2.93 | 5.73 | 4.93 | 7.23 | 6.73 | 4.43 |
| cj8 | 6.09 | 6.84 | 7.06 | 7.37 | 7.33 | 7.24 |  | 5.33 | 4.50 | 2.67 | 1.50 | 4.57 | 4.20 | 7.00 | 7.00 | 6.37 |
| cj9 | 4.92 | 6.53 | 5.97 | 6.62 | 7.32 | 7.00 |  | 3.47 | 4.09 | 3.53 | 3.17 | 6.00 | 5.37 | 6.86 | 6.29 | 2.93 |
| cj10 | 4.92 | 7.06 | 5.91 | 7.05 | 7.03 | 7.19 |  | 3.23 | 3.96 | 4.29 | 3.14 | 5.57 | 5.53 | 7.00 | 6.29 | 2.93 |
| cj11 | 4.84 | 7.09 | 5.82 | 6.57 | 6.93 | 6.88 |  | 3.27 | 4.83 | 4.23 | 3.43 | 6.57 | 5.04 | 6.76 | 6.37 | 2.83 |
| yj1 | 6.43 | 6.84 | 7.19 | 7.47 | 7.47 | 7.25 |  | 5.47 | 4.71 | 2.33 | 1.33 | 5.67 | 4.33 | 7.43 | 7.17 | 6.27 |
| qy1 | 6.57 | 6.84 | 7.57 | 7.69 | 7.53 | 7.10 |  | 5.76 | 4.43 | 2.43 | 1.27 | 5.33 | 4.00 | 7.53 | 7.27 | 6.33 |
| yj2 | 5.82 | 7.22 | 6.93 | 7.36 | 7.28 | 7.01 |  | 5.37 | 4.55 | 3.23 | 2.93 | 5.93 | 5.04 | 7.23 | 7.33 | 4.73 |
| cj12 | 5.15 | 6.63 | 5.87 | 7.01 | 7.37 | 7.06 |  | 3.29 | 4.19 | 4.00 | 2.27 | 6.83 | 5.43 | 7.53 | 6.43 | 2.86 |
| cj13 | 5.15 | 7.03 | 5.73 | 7.03 | 7.10 | 7.10 |  | 3.23 | 4.00 | 4.17 | 3.29 | 6.96 | 5.43 | 6.96 | 5.96 | 2.83 |
| cj14 | 4.72 | 7.00 | 6.13 | 6.57 | 6.79 | 6.93 |  | 3.19 | 3.83 | 4.27 | 3.50 | 5.53 | 5.47 | 6.97 | 6.00 | 2.67 |
| qt1 | 6.26 | 7.19 | 6.59 | 7.32 | 7.13 | 6.77 |  | 5.07 | 4.04 | 3.83 | 3.43 | 5.76 | 5.00 | 7.07 | 6.57 | 5.23 |
| yy2 | 5.97 | 7.47 | 6.93 | 7.47 | 7.37 | 7.06 |  | 5.50 | 4.27 | 2.20 | 2.27 | 5.27 | 4.76 | 7.23 | 7.00 | 4.50 |
| cy1 | 5.82 | 7.06 | 6.90 | 7.53 | 7.69 | 6.90 |  | 5.14 | 3.96 | 3.93 | 3.43 | 5.76 | 5.27 | 7.04 | 6.33 | 4.57 |
| yt1 | 6.34 | 6.65 | 7.06 | 7.50 | 7.47 | 7.10 |  | 5.43 | 4.71 | 2.50 | 1.29 | 5.53 | 4.14 | 7.57 | 7.27 | 6.33 |
| yj3 | 4.92 | 7.06 | 5.87 | 6.81 | 7.00 | 7.06 |  | 3.43 | 3.96 | 4.00 | 3.33 | 5.43 | 5.19 | 7.07 | 6.33 | 2.76 |
| yy3 | 6.72 | 7.22 | 7.10 | 7.69 | 7.73 | 7.47 |  | 5.76 | 4.23 | 2.23 | 1.17 | 5.03 | 4.03 | 7.53 | 7.14 | 6.37 |
| yy4 | 5.06 | 7.33 | 6.53 | 7.22 | 7.19 | 7.00 |  | 5.19 | 4.09 | 3.23 | 3.17 | 5.73 | 5.63 | 7.14 | 6.73 | 4.43 |
| yy5 | 5.99 | 7.47 | 6.59 | 7.32 | 7.15 | 7.13 |  | 4.63 | 4.33 | 3.73 | 3.33 | 6.33 | 5.33 | 7.23 | 6.67 | 4.53 |
| cj17 | 5.12 | 6.66 | 5.78 | 7.06 | 7.37 | 7.10 |  | 3.17 | 4.17 | 4.27 | 3.07 | 6.53 | 5.50 | 7.04 | 6.33 | 3.00 |
| yj4 | 4.50 | 7.19 | 6.59 | 7.37 | 6.59 | 6.81 |  | 4.93 | 3.93 | 3.50 | 3.29 | 6.73 | 5.33 | 7.14 | 6.43 | 4.53 |
| yq1 | 6.26 | 7.33 | 6.88 | 7.47 | 7.24 | 6.75 |  | 5.37 | 4.19 | 3.93 | 2.86 | 5.93 | 5.00 | 7.17 | 6.27 | 4.67 |
| cq2 | 5.78 | 6.62 | 5.82 | 7.10 | 7.24 | 7.28 |  | 3.19 | 4.23 | 3.96 | 3.14 | 7.24 | 5.34 | 7.27 | 6.97 | 3.07 |
| yt2 | 6.26 | 7.19 | 6.93 | 7.60 | 7.47 | 7.10 |  | 5.67 | 4.73 | 2.20 | 1.33 | 5.33 | 4.17 | 7.27 | 7.17 | 6.20 |
| yt3 | 6.19 | 7.47 | 7.37 | 7.37 | 7.37 | 7.15 |  | 5.43 | 3.57 | 2.43 | 1.57 | 4.80 | 4.29 | 7.29 | 7.17 | 6.57 |
| yy6 | 6.57 | 7.13 | 6.62 | 7.50 | 7.60 | 7.22 |  | 5.71 | 4.67 | 3.04 | 1.14 | 5.27 | 4.14 | 7.29 | 7.17 | 5.96 |
| cy2 | 5.03 | 6.43 | 5.90 | 7.00 | 7.01 | 7.03 |  | 3.27 | 3.93 | 3.97 | 3.23 | 6.76 | 5.43 | 6.83 | 6.37 | 2.57 |
| yy7 | 5.82 | 7.32 | 6.75 | 7.13 | 7.10 | 7.00 |  | 5.17 | 3.93 | 4.17 | 3.23 | 5.67 | 5.23 | 6.96 | 6.76 | 4.58 |
| tl1 | 6.06 | 7.33 | 6.75 | 7.10 | 7.19 | 7.19 |  | 4.96 | 4.07 | 3.50 | 3.23 | 5.67 | 5.23 | 7.17 | 6.43 | 4.73 |
| yj5 | 6.13 | 7.32 | 6.81 | 7.03 | 6.59 | 6.81 |  | 5.29 | 4.33 | 3.93 | 2.43 | 5.79 | 5.29 | 7.43 | 6.86 | 4.67 |
| qy2 | 6.34 | 7.37 | 6.93 | 7.60 | 7.57 | 7.22 |  | 5.33 | 4.54 | 2.53 | 1.43 | 5.93 | 4.19 | 7.27 | 6.97 | 6.50 |
| yj6 | 5.85 | 7.33 | 6.62 | 7.24 | 7.33 | 6.90 |  | 5.33 | 4.20 | 3.86 | 3.00 | 6.19 | 5.06 | 6.96 | 6.57 | 4.33 |
| yy8 | 6.34 | 7.22 | 7.33 | 7.60 | 7.47 | 7.37 |  | 5.57 | 4.51 | 2.33 | 1.37 | 5.37 | 4.14 | 7.43 | 7.33 | 6.53 |
| cy3 | 5.00 | 6.43 | 5.97 | 6.65 | 7.10 | 7.19 |  | 4.50 | 4.04 | 4.29 | 2.27 | 6.76 | 5.43 | 6.76 | 6.27 | 2.83 |
| yy9 | 6.40 | 6.90 | 7.06 | 7.47 | 7.37 | 6.59 |  | 5.53 | 4.63 | 2.27 | 1.29 | 5.17 | 4.14 | 7.27 | 7.14 | 6.33 |
| yq2 | 5.03 | 6.63 | 5.87 | 7.03 | 7.03 | 7.22 |  | 3.29 | 4.04 | 4.00 | 3.53 | 6.76 | 5.53 | 7.17 | 6.07 | 3.00 |
| yz1 | 6.13 | 7.06 | 6.84 | 7.37 | 7.33 | 6.90 |  | 5.20 | 4.27 | 3.53 | 3.33 | 5.50 | 5.17 | 7.27 | 6.43 | 4.76 |
| yj7 | 6.13 | 7.47 | 6.88 | 7.37 | 7.50 | 7.05 |  | 5.23 | 4.17 | 3.76 | 2.96 | 5.83 | 5.17 | 7.17 | 6.53 | 4.53 |
| yy10 | 6.47 | 7.41 | 7.06 | 7.37 | 7.53 | 7.19 |  | 5.80 | 4.53 | 2.96 | 1.29 | 4.76 | 4.33 | 7.23 | 7.27 | 6.23 |
| cy6 | 4.96 | 6.34 | 5.97 | 6.85 | 7.10 | 6.88 |  | 3.04 | 4.00 | 4.17 | 2.86 | 6.79 | 4.96 | 6.86 | 6.17 | 2.73 |
| yt4 | 5.50 | 7.06 | 6.75 | 6.75 | 6.53 | 6.81 |  | 5.00 | 4.00 | 3.76 | 3.14 | 5.53 | 5.27 | 7.07 | 6.33 | 4.63 |
| yy11 | 6.43 | 7.00 | 7.22 | 7.69 | 7.37 | 7.32 |  | 4.96 | 4.51 | 2.50 | 1.69 | 5.23 | 4.33 | 7.57 | 7.20 | 6.37 |
| yy12 | 5.87 | 6.72 | 7.06 | 7.73 | 7.69 | 7.37 |  | 5.67 | 4.47 | 2.53 | 1.23 | 5.76 | 4.27 | 7.53 | 7.27 | 6.43 |
| yq3 | 5.24 | 6.90 | 6.93 | 7.47 | 7.33 | 7.28 |  | 4.83 | 4.67 | 2.33 | 1.33 | 4.86 | 4.00 | 7.34 | 6.50 | 6.19 |
| cq1 | 5.59 | 7.00 | 6.43 | 6.90 | 6.90 | 7.03 |  | 4.71 | 3.76 | 4.17 | 3.57 | 5.79 | 5.37 | 6.93 | 5.96 | 4.53 |
| yq4 | 5.85 | 7.32 | 6.93 | 7.69 | 7.41 | 7.22 |  | 5.43 | 4.33 | 3.76 | 3.33 | 5.33 | 5.33 | 7.33 | 6.59 | 4.33 |
| cj15 | 4.96 | 6.44 | 5.87 | 6.92 | 6.90 | 7.06 |  | 3.07 | 3.96 | 4.04 | 3.14 | 6.96 | 5.33 | 6.83 | 5.96 | 3.33 |
| yy13 | 5.90 | 7.47 | 6.53 | 7.57 | 7.37 | 7.13 |  | 5.43 | 4.50 | 3.27 | 3.00 | 5.36 | 5.14 | 7.29 | 6.67 | 5.00 |
| yy14 | 5.97 | 7.06 | 6.90 | 7.33 | 7.37 | 7.19 |  | 5.43 | 4.37 | 3.43 | 3.17 | 6.33 | 4.96 | 7.43 | 6.47 | 4.33 |
| yy15 | 5.82 | 7.22 | 6.40 | 6.94 | 7.19 | 6.75 |  | 5.29 | 3.96 | 3.83 | 3.14 | 5.80 | 5.17 | 7.04 | 6.53 | 4.29 |
| yz2 | 7.19 | 7.47 | 7.10 | 7.32 | 7.69 | 7.53 |  | 5.67 | 4.53 | 2.33 | 1.53 | 4.93 | 3.27 | 7.57 | 7.19 | 6.04 |
| yz3 | 5.87 | 7.19 | 6.53 | 7.57 | 7.19 | 6.94 |  | 5.33 | 4.17 | 3.76 | 3.29 | 6.00 | 5.04 | 7.17 | 6.37 | 5.19 |
| cz2 | 4.92 | 6.09 | 5.99 | 6.75 | 7.03 | 7.00 |  | 3.17 | 4.00 | 4.07 | 3.43 | 6.86 | 5.37 | 6.87 | 5.83 | 2.63 |
| cj18 | 5.06 | 6.75 | 5.87 | 7.22 | 6.90 | 7.06 |  | 3.43 | 4.20 | 4.33 | 3.07 | 7.04 | 5.47 | 6.86 | 6.33 | 2.76 |
| cy4 | 5.06 | 6.57 | 5.99 | 6.90 | 7.28 | 7.06 |  | 3.90 | 4.00 | 4.14 | 3.33 | 6.58 | 5.43 | 7.23 | 6.23 | 3.17 |
| yy16 | 4.90 | 7.33 | 6.88 | 7.15 | 7.37 | 6.81 |  | 5.29 | 4.04 | 4.00 | 3.50 | 5.64 | 5.17 | 7.04 | 7.19 | 4.27 |
| cy5 | 6.09 | 7.47 | 6.66 | 7.69 | 7.29 | 7.10 |  | 5.23 | 4.43 | 3.29 | 3.07 | 6.14 | 5.04 | 7.27 | 6.57 | 4.23 |
| yz4 | 5.99 | 7.47 | 6.66 | 7.19 | 7.53 | 7.19 |  | 5.29 | 4.50 | 2.29 | 3.27 | 5.83 | 5.27 | 7.43 | 6.53 | 4.53 |
| yy17 | 6.09 | 7.10 | 6.79 | 7.19 | 7.60 | 7.13 |  | 5.27 | 4.50 | 2.27 | 2.96 | 5.96 | 5.09 | 7.33 | 6.86 | 4.29 |
| yy18 | 6.28 | 7.10 | 6.66 | 7.47 | 7.28 | 7.10 |  | 5.33 | 4.29 | 2.43 | 1.33 | 5.27 | 4.29 | 7.33 | 7.00 | 6.33 |
| yq5 | 5.82 | 7.06 | 6.75 | 7.19 | 7.22 | 7.15 |  | 4.67 | 4.07 | 4.00 | 3.04 | 6.07 | 4.96 | 7.14 | 6.43 | 4.57 |
| yy19 | 6.66 | 7.06 | 7.22 | 7.60 | 7.69 | 7.37 |  | 5.86 | 4.57 | 2.29 | 1.27 | 4.96 | 4.85 | 7.14 | 7.00 | 5.33 |
| yy20 | 6.09 | 6.93 | 6.93 | 7.42 | 7.53 | 7.32 |  | 5.43 | 4.33 | 2.34 | 2.67 | 5.63 | 4.27 | 7.27 | 7.14 | 6.37 |
| ct1 | 5.50 | 6.15 | 6.06 | 7.03 | 7.10 | 7.19 |  | 3.00 | 4.27 | 2.86 | 3.07 | 6.93 | 5.33 | 7.23 | 6.33 | 3.14 |
| yy21 | 5.71 | 7.22 | 6.53 | 7.32 | 7.33 | 7.15 |  | 5.23 | 4.29 | 3.43 | 2.37 | 5.69 | 5.19 | 7.29 | 6.43 | 4.29 |
| cz1 | 5.66 | 6.72 | 5.90 | 6.68 | 7.24 | 7.13 |  | 3.33 | 4.19 | 4.23 | 3.04 | 6.83 | 5.17 | 7.00 | 6.33 | 2.23 |
| ct2 | 4.92 | 6.53 | 5.97 | 6.82 | 7.03 | 6.90 |  | 3.47 | 4.00 | 4.04 | 3.29 | 6.79 | 5.43 | 6.93 | 6.00 | 2.67 |
| yy22 | 6.53 | 7.00 | 7.19 | 7.53 | 7.73 | 7.13 |  | 5.20 | 4.50 | 2.27 | 1.27 | 4.80 | 4.17 | 7.17 | 7.14 | 5.00 |
| cj19 | 5.23 | 6.81 | 5.78 | 7.18 | 7.37 | 7.41 |  | 3.33 | 4.19 | 4.04 | 2.96 | 6.19 | 4.33 | 7.33 | 6.53 | 3.17 |
| yy23 | 6.43 | 7.10 | 6.90 | 7.53 | 7.69 | 7.22 |  | 4.48 | 4.43 | 2.96 | 2.07 | 5.17 | 4.27 | 7.37 | 7.29 | 5.63 |
| yq6 | 6.26 | 7.37 | 6.66 | 7.29 | 7.37 | 7.15 |  | 5.27 | 4.33 | 3.73 | 2.23 | 5.47 | 5.17 | 7.23 | 7.00 | 4.33 |
| yq7 | 5.82 | 7.13 | 6.53 | 7.13 | 7.33 | 7.28 |  | 5.23 | 4.00 | 3.83 | 3.04 | 5.57 | 5.14 | 7.23 | 6.67 | 4.43 |
| qy4 | 5.82 | 7.53 | 6.90 | 7.47 | 7.59 | 7.32 |  | 5.54 | 4.33 | 2.67 | 2.93 | 5.86 | 5.50 | 7.37 | 7.04 | 4.58 |
| yt5 | 4.92 | 7.47 | 6.75 | 7.47 | 7.37 | 7.28 |  | 5.43 | 4.27 | 4.01 | 2.24 | 6.17 | 5.22 | 7.50 | 6.57 | 4.63 |
| yj8 | 5.66 | 6.81 | 6.53 | 7.47 | 7.57 | 7.19 |  | 5.37 | 4.43 | 4.07 | 1.92 | 5.83 | 5.33 | 7.33 | 6.67 | 4.83 |
| yy24 | 5.97 | 6.90 | 6.93 | 7.32 | 7.47 | 7.37 |  | 5.39 | 4.43 | 3.33 | 2.86 | 6.50 | 4.96 | 7.17 | 6.93 | 5.19 |
| yz5 | 5.99 | 7.32 | 6.79 | 7.41 | 7.32 | 7.03 |  | 5.20 | 4.76 | 2.83 | 3.04 | 5.76 | 5.14 | 7.14 | 6.53 | 3.63 |
| yy25 | 5.87 | 7.37 | 6.90 | 7.50 | 7.47 | 7.15 |  | 4.96 | 4.67 | 3.07 | 2.83 | 6.00 | 5.17 | 7.23 | 7.19 | 4.73 |
| qy3 | 6.34 | 6.62 | 7.32 | 7.32 | 7.47 | 7.19 |  | 5.14 | 4.50 | 2.50 | 1.27 | 4.83 | 3.93 | 7.37 | 6.27 | 6.37 |
| yt6 | 6.13 | 7.37 | 6.94 | 7.37 | 7.37 | 6.96 |  | 5.23 | 4.23 | 4.17 | 3.27 | 5.85 | 5.20 | 7.43 | 6.43 | 4.62 |
| yq8 | 5.82 | 6.75 | 7.00 | 7.19 | 7.19 | 7.10 |  | 5.17 | 4.27 | 4.58 | 3.43 | 5.93 | 4.93 | 7.23 | 6.47 | 3.53 |
| cj16 | 5.12 | 6.59 | 5.97 | 7.00 | 7.60 | 7.03 |  | 3.37 | 4.00 | 3.96 | 2.89 | 7.00 | 5.47 | 7.19 | 6.13 | 2.93 |
| cy7 | 5.90 | 7.37 | 6.75 | 7.37 | 7.47 | 7.22 |  | 5.29 | 4.43 | 3.93 | 3.04 | 5.83 | 4.93 | 7.37 | 7.30 | 4.23 |
| yy26 | 6.28 | 7.53 | 6.79 | 7.38 | 7.33 | 7.47 |  | 5.43 | 4.67 | 2.19 | 1.53 | 5.19 | 4.00 | 7.53 | 7.23 | 6.14 |

Table S3 Cluster centroid result of jasmine tea for the aroma factor

| Category | Freshness | Consistence | Persistence | Purity | Concentration | Strength |
| --- | --- | --- | --- | --- | --- | --- |
| FL | 6.35±0.33 | 7.08±0.27 | 7.07±0.21 | 7.5±0.12 | 7.5±0.14 | 7.24±0.18 |
| HT | 5.04±0.3 | 6.62±0.27 | 5.89±0.1 | 6.9±0.19 | 7.13±0.19 | 7.07±0.13 |
| FH | 5.81±0.38 | 7.24±0.19 | 6.73±0.16 | 7.29±0.21 | 7.26±0.27 | 7.04±0.17 |

Note: The underline indicates that the factor of this kind of jasmine tea has the highest score.

Table S4 Cluster centroid result of jasmine tea for the taste factor

| Category | Freshness | Sweet | Bitter | Astringent | Dense | Thick | Purity | Coordination | Floral |
| --- | --- | --- | --- | --- | --- | --- | --- | --- | --- |
| FL | 5.43 | 4.49 | 2.49 | 1.44 | 5.19 | 4.13 | 7.37 | 7.08 | 6.18 |
| HT | 3.35 | 4.08 | 4.03 | 3.15 | 6.60 | 5.34 | 7.03 | 6.26 | 2.88 |
| FH | 5.20 | 4.23 | 3.60 | 3.02 | 5.84 | 5.16 | 7.21 | 6.64 | 4.52 |

Note: The underline indicates that the factor of this kind of jasmine tea has the highest score.

Table S5 OAV value of common aroma components in different types of jasmine tea

| NO. | Name | OAV | | |
| --- | --- | --- | --- | --- |
|  |  | FL | HT | FH |
| 1 | Linalool | 35.80×10^3^ | 10.42×10^3^ | 14.90×10^3^ |
| 2 | Acetic acid phenylmethyl ester | 29.26×10^3^ | 17.38×10^3^ | 20.54×10^3^ |
| 3 | Nerolidol | 267.27 | 157.10 | 124.90 |
| 4 | Benzoic acid methyl ester | 246.64 | 61.51 | 93.30 |
| 5 | (E)-2-Methyl-propanoic acid-3,7-dimethyl-2,6-octadienyl ester | 218.72 | - | - |
| 6 | Indole | 132.01 | 111.54 | 105.41 |
| 7 | Methyl salicylate | 118.93 | 43.81 | 53.41 |
| 8 | Decanal | 83.38 | 77.47 | 65.60 |
| 9 | Geraniol | 78.82 | 49.59 | 48.91 |
| 10 | 2-(Methylamino)-benzoic acid methyl ester | 53.70 | 28.93 | 28.87 |
| 11 | Farnesol | 33.14 | - | - |
| 12 | 6-Methyl-5-hepten-2-one | 29.67 | - | - |
| 13 | Trans-linalool oxide (furanoid) | 28.77 | - | - |
| 14 | Linalool oxide II (pyran) | 20.98 | - | 11.12 |
| 15 | l-Caryophyllene | 19.16 | 5.09 | 6.11 |
| 16 | β-Myrcene | 17.89 | 7.56 | 9.61 |
| 17 | Acetic acid-2-phenylethyl ester | 12.23 | 11.85 | 11.26 |
| 18 | β-cyclocitral | 12.13 | 13.84 | 13.78 |
| 19 | Benzoic acid ethyl ester | 7.27 | 2.18 | 2.77 |
| 20 | (E)-3-Hexen-1-ol | 6.65 | 3.15 | 4.11 |
| 21 | Benzyl alcohol | 1.61 | 0.77 | 0.62 |
| 22 | Benzaldehyde | 1.06 | 0.61 | 0.55 |
| 23 | Hexanal | 15.51 | - | - |
| 24 | (Z)-3-Hexen-1-ol acetate | - | - | 70.95 |
| 25 | 2,6-Dimethyl-5-heptenal | 116.95 | 87.54 | - |
| 26 | Dodecanal | - | 19.61 | - |
| 27 | Nonanal | 53.78 | 48.29 | 41.17 |
| 28 | Linalool oxide Ⅲ (pyran) | - | 13.45 | - |
| 29 | Geranyl acetate | - | - | 15.57 |
| 30 | beta-Ionone | - | 5.91×10^3^ | - |

Note: “-” indicates no data.
